# On Hill et al's conjecture for calculating the subtree prune and regraft distance between phylogenies

**DOI:** 10.1186/1471-2148-10-334

**Published:** 2010-10-29

**Authors:** Simone Linz

**Affiliations:** 1 Department of Computer Science, Technical University of Catalonia, Barcelona, Spain

## Abstract

**Background:**

Recently, Hill et al. [1] implemented a new software package--called SPRIT--which aims at calculating the minimum number of horizontal gene transfer events that is needed to simultaneously explain the evolution of two rooted binary phylogenetic trees on the same set of taxa. To this end, SPRIT computes the closely related so-called rooted subtree prune and regraft distance between two phylogenies. However, calculating this distance is an NP-hard problem and exact algorithms are often only applicable to small- or medium-sized problem instances. Trying to overcome this problem, Hill et al. propose a divide-and-conquer approach to speed up their algorithm and conjecture that this approach can be used to compute the rooted subtree prune and regraft distance exactly.

**Results:**

In this note, we present a counterexample to Hill et al's conjecture and subsequently show that a modified version of their conjecture holds.

**Conclusion:**

While Hill et al's conjecture may result in an overestimate of the rooted subtree prune and regraft distance, a slightly more restricted version of their approach gives the desired outcome and can be applied to speed up the exact calculation of this distance between two phylogenies.

## Background

In recent years, one of the main research foci in the development of theoretical frameworks that aim at approaching questions in evolutionary biology turns from the reconstruction of phylogenetic trees towards the reconstruction of phylogenetic networks. This has partly been triggered by the exponentially growing amount of available sequence data arising from whole genome sequencing projects and a successive detection of genes whose sequences are chimeras of distinct ancestral gene sequences, and hence, are likely to be the result of reticulation (e.g. horizontal gene transfer or hybridization). Although evolutionary biologists are now mostly acknowledging the existence of species arising from reticulation within certain groups of organisms, the extent to which such events have influenced the evolutionary history for a set of present-day species remains controversially discussed until today. To shed light on this question, Hill et al. [1] recently published a study that is centered around the identification and quantification of horizontal gene transfer. The authors have implemented a new software package--called SPRIT--consisting of a heuristic as well as an exact algorithm, applied it to several data sets of variable size, and compared their results and running times with those obtained from other algorithms that have previously been developed to analyze reticulate evolution.

Algorithmically, SPRIT draws on ideas that are borrowed from work that has been done in the context of the graph-theoretic operation of rooted subtree prune and regraft (rSPR) which is a popular tool to quantify the dissimilarity between two trees. Loosely speaking, an rSPR operation cuts (prunes) a subtree and reattaches (regrafts) it to another part of the tree. A lower bound on the number of reticulation events that is needed to simultaneously explain two phylogenies is the minimum number of rSPR operations that transform one phylogeny into the other [2,3]. This minimum number, which is computed by SPRIT, is referred to as the rSPR distance. However, since the task of calculating this distance is an NP-hard optimization problem, the application of exact algorithms is often restricted to medium-sized data sets.

In trying to overcome this obstacle, thus to speed up SPRIT, Hill et al. propose a divide-and-conquer-type reduction that breaks the problem into several smaller and more tractable subproblems before calculating the rSPR distance for each subproblem separately. Briefly, the authors conjecture that the sum of rSPR distances over all smaller subproblems is equal to the rSPR distance of the original unreduced trees. In this note, we give a counterexample to their conjecture. Nevertheless, we subsequently show that a slightly more restricted version of their conjecture holds and can be used to exactly calculate the rSPR distance between two phylogenies by breaking the problem into smaller subproblems.

The remainder of this paper is organized as follows. The next section contains some mathematical preliminaries that are needed to formally state Hill at al's conjecture. This conjecture is then given in the subsequent section which also contains the aforementioned counterexample. We then show that a modified version of the conjecture holds in the following section. We end this note with a brief conclusion.

## Preliminaries

In this section, we give some preliminary definitions that are used throughout this paper. Unless otherwise stated, the notation and terminology follows [4].

### Phylogenetic Trees

A *rooted binary phylogenetic X-tree *T is a rooted tree whose root has degree two while all other interior vertices have degree three and whose leaf set is *X *. The set *X *is the *label set *of T and is frequently denoted by ℒ(T). Furthermore, let *X*′ be a subset of *X*. The *minimal rooted subtree *of T that connects all the leaves in *X*′ is denoted by T(*X*′) while the *restriction of *T*to X*′, denoted by T|*X*′, is the rooted binary phylogenetic *X*′-tree obtained from T(*X*′) by contracting all degree-two vertices apart from the root.

### Rooted Subtree Prune and Regraft

Let T be a rooted binary phylogenetic *X*-trees. For the purposes of the upcoming definition, we view the root of T as a vertex *ρ *adjoined to the original root by a pendant edge. Now, let *e *= {*u*, *v*} be any edge of T that is not incident with *ρ *such that *u *is the vertex on the path from *ρ *to v. Let $T^{\prime }$ be the rooted binary phylogenetic *X*-tree obtained from T by deleting *e *and reattaching the resulting subtree with root *v *via a new edge, say *f *, as follows. Subdivide an edge of the component that contains *ρ *with a new vertex *u′*, join *u′ *and v with *f *, and contract *u*. Then $T^{\prime }$ has been obtained from T by a *rooted subtree prune and regraft (rSPR) operation*. The rSPR *distance *between two rooted binary phylogenetic *X*-trees T and $T^{\prime }$ is the minimum number of rSPR operations that transform T into $T^{\prime }$. We denote this distance by $d_{\text{rSPR}}(T,T^{\prime })$.

### Agreement Forests

Let T and $T^{\prime }$ be two rooted binary phylogenetic *X*-trees. Again, to make the following work, regard the roots of T and $T^{\prime }$ as a vertex *ρ *adjoined to the original root by a pendant edge. An *agreement forest *$ℱ=\{{ℒ}_{p},{ℒ}_{1},{ℒ}_{2},...,{ℒ}_{k}\}$ for T and $T^{\prime }$ is a partition of X∪{ρ} such that $\rho \in {ℒ}_{\rho }$ and the following properties are satisfied:

(i) for all *i *∈ {*ρ*, 1, ..., *k*}, we have $T|{ℒ}_{i}≅T^{\prime }\text{|}{ℒ}_{i}$, and

(ii) the trees in $\left\{T({ℒ}_{i}):i\in \{\rho ,1,...,k\}\right\}$ and $\left\{T^{\prime }({ℒ}_{i}):i\in \{\rho ,1,...,k\}\right\}$ are vertex-disjoint subtrees of T and $T^{\prime }$, respectively.

Throughout the remainder of this note, we will interchangeably refer to $\left\{T|{ℒ}_{\rho },T|{ℒ}_{1},T|{ℒ}_{2},...,T|{ℒ}_{k}\right\}$ and $\left\{{ℒ}_{\rho },{ℒ}_{1},...,{ℒ}_{k}\right\}$ as an agreement forest for T and $T^{\prime }$. A *maximum-agreement forest *for T and $T^{\prime }$ is an agreement forest for T and $T^{\prime }$ with the smallest number of elements over all agreement forests for T and $T^{\prime }$. Note that a maximum-agreement forest for T and $T^{\prime }$ is not necessarily unique.

Bordewich and Semple [5] established the following characterization which directly relates the rSPR distance to the number of elements in a maximum-agreement forest and is crucial to many algorithms that exactly compute the rSPR distance between two rooted binary phylogenetic trees.

**Theorem 1**. *Let *T*and *$T^{\prime }$*be two rooted binary phylogenetic X-trees, and let *$\left\{T_{\rho },T_{1},T_{2},...,T_{k}\right\}$*be a maximum-agreement forest for *T*and *$T^{\prime }$*. Then*

$$d_{\text{rSPR}}(T,T^{\prime })=k.$$

### Clusters

Let T be a rooted binary phylogenetic *X*-tree, and let *A *be a subset of *X *with |*A*| ≥ 2. We say that *A *is a *cluster *of T if there is a vertex v in T whose set of descendants is precisely *A*. We denote this cluster by $C_{T}(v)$.

**Figure 1:**
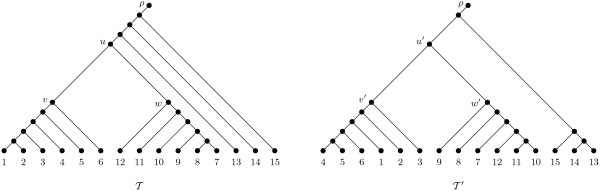
**Two rooted binary phylogenetic *X*-trees T and $T^{\prime }$**. Note that T and $T^{\prime }$ have an additional vertex *ρ *adjoined to the original root by a pendant edge.

We next consider several different types of clusters that will play an important role in the remainder of this paper. Let T and $T^{\prime }$ be two rooted binary phylogenetic *X*-trees, and let *A *be a cluster that is common to T and $T^{\prime }$; that is there exists a vertex v in T and a vertex $v^{\prime }$ in $T^{\prime }$ such that $C_{T}(v)=C_{T^{\prime }}(v^{\prime })$. Furthermore, let *u *(resp. *u′*) be the parent vertex of v (resp. $v^{\prime }$) in T (resp. $T^{\prime }$), and let *w *(resp. *w′*) be the child vertex of *u *(resp. *u′*) with w≠v (resp. $w^{\prime }\neq v^{\prime }$). If no proper subset of *A *is a common cluster of T and $T^{\prime }$, we refer to *A *as a *minimal cluster*. Moreover, *A *is a *solvable cluster *if *A *is minimal and $C_{T}(u)=C_{T^{\prime }}(u^{\prime })$. Lastly, we say that *A *is a *subtree-like cluster *if *A *is a solvable cluster and $T\text{|}C_{T}(w)≅T^{\prime }\text{|}C_{T^{\prime }}(w^{\prime })$. Roughly speaking, the condition $T\text{|}C_{T}(w)≅T^{\prime }\text{|}C_{T^{\prime }}(w^{\prime })$ is satisfied if the subtree with root *w *in T is identical to the subtree with root *w′ *in $T^{\prime }$. We refer to $T\text{|}C_{T}(w)$ as the *common subtree associated with A *and note that it can exclusively consist of an isolated vertex. For example, *A *= {1, 2, ..., 6} is a solvable cluster of the two rooted binary phylogenetic *X*-trees T and $T^{\prime }$ that are shown in Figure 1 since $C_{T}(u)=C_{T^{\prime }}(u^{\prime })$ = {1, 2, ..., 12}. However, as T|(7,8, …, 12)≅T′|(7,8, …, 12), it follows that *A *is not a subtree-like cluster of T and $T^{\prime }$.

**Figure 2:**
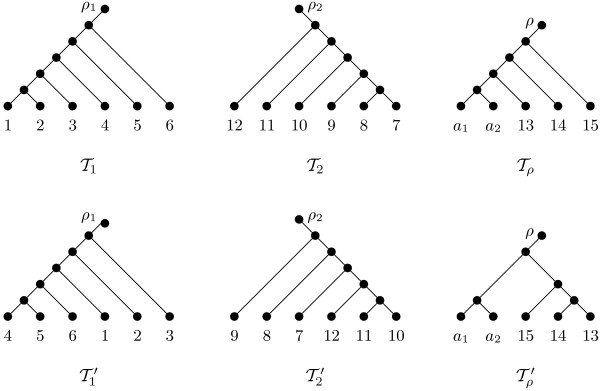
**A cluster sequence with respect to Θ = *solvable *for the two rooted binary phylogenetic *X*-trees T and $T^{\prime }$ shown in Figure 1**. Details on how the tree pairs have been obtained are given in the text.

Now, let Θ ∈ {*minimal, solvable, subtree-like*}. We next describe algorithmically how to obtain a sequence of tree pairs--which is important to mathematically state Hill et al's conjecture--by decomposing two rooted binary phylogenetic *X*-trees T and $T^{\prime }$ into smaller subtrees. As previously, view the roots of T and $T^{\prime }$ as a vertex *ρ *adjoined to the original root by a pendant edge, and regard *ρ *as part of the label set; that is ℒ(T)=X∪{ρ}. Setting *i *to be 1, let *A_i _*be a common Θ cluster of T and $T^{\prime }$ with $|ℒ(T)|-|A_{i}|>1$. Let $T_{i}$ denote the rooted binary phylogenetic tree $T|A_{i}$ (viewing the root of $T_{i}$ as a vertex *ρ_i _*adjoined to the original root by a pendant edge) and reset T to be the tree obtained from T by replacing $T(A_{i})$ with a new vertex *a_i _*. Analogously, let ${T^{\prime }}_{i}$ denote the rooted binary phylogenetic tree $T^{\prime }|A_{i}$ (viewing the root of ${T^{\prime }}_{i}$ as a vertex *ρ_i _*adjoined to the original root by a pendant edge) and reset $T^{\prime }$ to be the tree obtained from $T^{\prime }$ by replacing $T^{\prime }(A_{i})$ with a new vertex *a_i _*. If T and $T^{\prime }$ contain a Θ cluster *A*_*i*+1 _with $|ℒ(T)|-|A_{i+1}|>1$, stop or increment *i *by 1 and repeat this process; otherwise, stop. Eventually, we obtain a sequence

$$\left(T_{\text{1}},{T^{\prime }}_{1}),...,(T_{t},{T^{\prime }}_{t}),(T_{\rho },{T^{\prime }}_{\rho }\right)$$

of pairs of rooted binary phylogenetic trees, where $T_{\rho }$ and ${T^{\prime }}_{\rho }$ denote the two trees after the replacement of $T(A_{t})$ and $T^{\prime }(A_{t})$ with a vertex *a_t_*. We call this sequence a *cluster sequence *of T and $T^{\prime }$ with respect to a specific cluster type Θ. An example of a cluster sequence with respect to Θ = *solvable *for the two rooted binary phylogenetic trees depicted in Figure 1 is shown in Figure 2.

## Hill et al's Conjecture and a Counterexample

We begin this section by formally stating Hill et al's conjecture which was introduced in [1].

**Conjecture 2**. *Let *T*and *$T^{\prime }$*be two rooted binary phylogenetic X-trees. Let *$\left(T_{\text{1}},{T^{\prime }}_{1}),...,(T_{t},{T^{\prime }}_{t}),(T_{\rho },{T^{\prime }}_{\rho }\right)$*be a cluster sequence for *T*and *$T^{\prime }$*with respect to *Θ = *solvable. Then*

(1) $$d_{\text{rSPR}}(T,T^{\prime })=\sum_{i=1}^{t}d_{\text{rSPR}}(T_{i},{T^{\prime }}_{i})+d_{\text{rSPR}}(T_{\rho },{T^{\prime }}_{\rho }).$$

**Figure 3:**
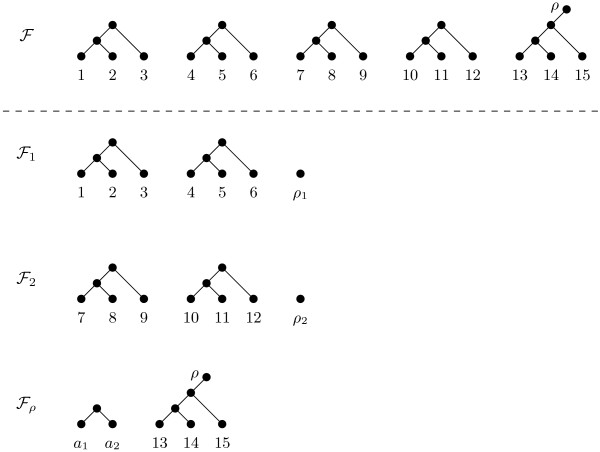
**Maximum-agreement forests**. Top: A maximum-agreement forest ℱ for T and $T^{\prime }$ depicted in Figure 1. Bottom: A maximum-agreement forest ${ℱ}_{i}$ for each tree pair $T_{i}$ and ${T^{\prime }}_{i}$ shown in Figure 2.

Next, we detail a counterexample to the above conjecture which is based on the two rooted binary phylogenetic *X*-trees T and $T^{\prime }$ that are shown in Figure 1. A maximum-agreement forest ℱ for T and $T^{\prime }$ contains 5 elements and is shown in the top of Figure 3. By Theorem 1, this implies that $d_{\text{rSPR}}(T,T^{\prime })=4$. Now, consider the cluster sequence with respect to Θ = *solvable *for T and $T^{\prime }$ that contains three tree pairs and is depicted in Figure 2. The first tree pair ($T_{1},{T^{\prime }}_{1}$) consists of the restricted subtrees of T and $T^{\prime }$ whose leaf set is the solvable cluster *A*_1 _= {1, 2, ..., 6} of T and $T^{\prime }$; thus $T_{1}=T|(A_{1}\cup \{\rho_{1}\})$ and ${T^{\prime }}_{1}=T^{\prime }|(A_{1}\cup \{\rho_{1}\})$. Similarly, the second tree pair ($T_{2},{T^{\prime }}_{2}$) consists of the restricted subtrees of the two trees that have been obtained from T and $T^{\prime }$ by replacing $T(A_{1})$ and $T^{\prime }(A_{1})$, respectively, with a single leaf *a*_1 _whose leaf set is the solvable cluster *A*_2 _= {7, 8, ..., 12}. Lastly, the third tree pair ($T_{\rho },{T^{\prime }}_{\rho }$) can be regarded as being obtained from T and $T^{\prime }$ by replacing $T(A_{1})$ and $T^{\prime }(A_{1})$ with a leaf *a*_1 _and replacing $T(A_{2})$ and $T^{\prime }(A_{2})$ with a leaf *a*_2_. For each tree pair ($T_{i},{T^{\prime }}_{i}$) of the cluster sequence shown in Figure 2, a maximum-agreement forest ${ℱ}_{i}$ with *i *∈ {1, 2, *ρ*} is depicted in the bottom part of Figure 3. Note that each forest ${ℱ}_{i}$ is the unique maximum-agreement forest for $T_{i}$ and ${T^{\prime }}_{i}$ Now, by Equation 1, we have

$$d_{\text{rSPR}}(T_{1},{T^{\prime }}_{1})+d_{\text{rSPR}}(T_{2},{T^{\prime }}_{2})+d_{\text{rSPR}}(T_{\rho },{T^{\prime }}_{\rho })=\text{2}+\text{2}+1=\text{5}$$

which is strictly greater than $d_{\text{rSPR}}(T,T^{\prime })$; thus showing that Conjecture 2 does not hold.

## Using Subtree-Like Clusters to Prove Hill et al's Conjecture

In this section, we show that Conjecture 2 holds, if we consider a subtree-like cluster instead of a solvable cluster in each iteration of computing a cluster sequence for two rooted binary phylogenetic trees. We first prove the result for a cluster sequence of size two and then see that this result generalizes to cluster sequences of greater size.

**Lemma 3**. *Let *T*and *$T^{\prime }$*be two rooted binary phylogenetic X-trees. Let *($T_{1},{T^{\prime }}_{1}$), ($T_{\rho },{T^{\prime }}_{\rho }$) *be a cluster sequence for *T*and *$T^{\prime }$*with respect to *Θ = *subtree-like. Then*

$$d_{\text{rSPR}}\left(T,T^{\prime }\right)=d_{\text{rSPR}}\left(T_{1},{T^{\prime }}_{1}\right)+d_{\text{rSPR}}(T_{\rho },{T^{\prime }}_{\rho }).$$

*Proof*. Let *A*_1 _be the subtree-like cluster $ℒ(T_{1})-\{\rho_{1}\}$ of T and $T^{\prime }$. We start by making an observation that is crucial for what follows. By the definition of a subtree-like cluster, there exists a common subtree, say S, that is associated with *A*_1 _in T and $T^{\prime }$. Clearly, S is also a common subtree of $T_{\rho }$ and ${T^{\prime }}_{\rho }$. Furthermore, as $T_{\rho }$ has been obtained from T by replacing $T(A_{1})$ with a single vertex *a*_1 _and as ${T^{\prime }}_{\rho }$ has been obtained from $T^{\prime }$ by replacing $T^{\prime }(A_{1})$ with a single vertex *a*_1_, it is easily checked that T|($ℒ(S)\cup \{a_{1}\}$) is a common subtree of $T_{\rho }$ and ${T^{\prime }}_{\rho }$.

We now show that

(2) $$d_{\text{rSPR}}\left(T,T^{\prime }\right)\leq d_{\text{rSPR}}\left(T_{1},{T^{\prime }}_{1}\right)+d_{\text{rSPR}}(T_{\rho },{T^{\prime }}_{\rho }).$$

Let ${ℱ}_{1}$ be a maximum-agreement forest for $T_{1}$ and ${T^{\prime }}_{1}$, and let ${ℱ}_{\rho }$ be a maximum-agreement forest for $T_{\rho }$ and ${T^{\prime }}_{\rho }$. By the observation prior to this paragraph, it follows from Proposition 3.2 of [5] that $ℒ(S)\cup \{a_{1}\}$ is a subset of an element, say ${ℒ}_{a_{1}}$, in ${ℱ}_{\rho }$. Furthermore, let ${ℒ}_{\rho_{1}}$ be the label set of ${ℱ}_{1}$ with *ρ*_1 _∈ ${ℒ}_{\rho_{1}}$. As ${ℱ}_{1}$ is an agreement forest for $T_{1}$ and ${T^{\prime }}_{1}$ and as ${ℱ}_{\rho }$ is such a forest for $T_{\rho }$ and ${T^{\prime }}_{\rho }$, it follows that

$$ℱ=({ℱ}_{1}\cup {ℱ}_{\rho }-\{{ℒ}_{\rho_{1}},\;{ℒ}_{a_{1}}\})\cup \{({ℒ}_{\rho_{1}}-\{\rho_{1}\})\cup ({ℒ}_{a_{1}}-\{a_{1}\})\}$$

is an agreement forest for T and $T^{\prime }$. As ${ℒ}_{a_{1}}$ - {*a*_1_} always contains an element, note that $\left({ℒ}_{\rho_{1}}-\{\rho_{1}\})\cup ({ℒ}_{a_{1}}-\{a_{1}\}\right)$ is never the empty set. Thus $|ℱ|=|{ℱ}_{1}|+|{ℱ}_{\rho }|-1$ and, by Theorem 1, we have

$$d_{\text{rSPR}}(T_{1},\;T_{1})+d_{\text{rSPR}}(T_{\rho },\;{T^{\prime }}_{\rho })=|{ℱ}_{1}|-1+|{ℱ}_{\rho }|-1=|ℱ|-1\geq d_{\text{rSPR}}(T,\;T^{\prime }).$$

This establishes Equation 2.

We now turn to the second part of this proof and show that

(3) $$d_{\text{rSPR}}(T,\;T^{\prime })\geq d_{\text{rSPR}}(T_{1},{T^{\prime }}_{1})+d_{\text{rSPR}}(T_{\rho },\;{T^{\prime }}_{\rho }).$$

Let ℱ be a maximum-agreement forest for T and $T^{\prime }$. The remainder of this part splits into two cases. First, assume that there exists an element in ℱ, say ${ℒ}_{m}$, such that ${ℒ}_{m}\cap A_{1}\neq \emptyset$ and ${ℒ}_{m}\cap (X-A_{1})\cup \{\rho \}\neq \emptyset$. Note that ${ℒ}_{m}$ is the only label set with the described properties, as otherwise, ℱ is not an agreement forest for T and $T^{\prime }$. Let ${ℒ}_{m^{\prime }}=({ℒ}_{m}\cap A_{1})\cup \{\rho_{1}\}$, and let ${ℒ}_{m^{''}}=({ℒ}_{m}\cap ((X-A_{1})\cup \{\rho \}))\cup \{a_{1}\}$. Since ℱ is an agreement forest for T and $T^{\prime }$,

$${ℱ}_{1}=\{ℒ\in ℱ:ℒ\subseteq A_{1}\}\cup \{{ℒ}_{m^{\prime }}\}$$

is such a forest for $T_{1}$ and ${T^{\prime }}_{1}$ and

$${ℱ}_{\rho }=\{ℒ\in ℱ:ℒ\subseteq ((X-A_{1})\cup \{\rho \})\}\cup \{{ℒ}_{m^{''}}\}$$

is an agreement forest for $T_{\rho }$ and ${T^{\prime }}_{\rho }$. Second, assume that no such element ${ℒ}_{m}$ exists. Hence, every element ℒ in ℱ is either a subset of *A*_1 _or a subset of $\left(X-A_{1})\cup \{\rho \right\}$. Furthermore, as *A*_1 _is a subtree-like cluster of T and $T^{\prime }$ whose associated common subtree is S, it again follows from Proposition 3.2 of [5], that ℒ(S) is a subset of an element, say ${ℒ}_{S}$, in ℱ. Now, as ℱ is an agreement forest for T and $T^{\prime }$, it follows that

$${ℱ}_{1}=\{ℒ\in ℱ:ℒ\subseteq A_{1}\}\cup \{\{\rho_{1}\}\}$$

is an agreement forest for $T_{1}$ and ${T^{\prime }}_{1}$ and

$${ℱ}_{\rho }=(\{ℒ\in ℱ:ℒ\subseteq ((X-A_{1})\cup \{\rho \})\}-\{{ℒ}_{S}\})\cup \{{ℒ}_{S}\cup \{a_{1}\}\}$$

is such a forest for $T_{\rho }$ and ${T^{\prime }}_{\rho }$. Regardless of whether or not ${ℒ}_{m}$ exists, we have $|ℱ|=|{ℱ}_{1}|+|{ℱ}_{\rho }|-1$, and therefore,

$$d_{\text{rSPR}}(T,T^{\prime }\;)=|ℱ|-1=|{ℱ}_{1}|+|{ℱ}_{\rho }|-2\geq d_{\text{rSPR}}(T_{1},\;{T^{\prime }}_{1})+d_{\text{rSPR}}(T_{\rho },\;{T^{\prime }}_{\rho }).$$

This establishes Equation 3, and combining Equations 2 and 3 completes the proof of this lemma.

The next theorem directly follows from repeated applications of Lemma 3.

**Theorem 4**. *Let *T*and *$T^{\prime }$*be two rooted binary phylogenetic X-trees. Let $\left(T_{\text{1}},{T^{\prime }}_{1}),...,(T_{t},{T^{\prime }}_{t}),(T_{\rho },{T^{\prime }}_{\rho }\right)$ be a cluster sequence for *T*and *$T^{\prime }$*with respect to *Θ = *subtree-like. Then*

$$d_{\text{rSPR}}(T,T^{\prime })=\sum_{i=1}^{t}d_{\text{rSPR}}(T_{i},{T^{\prime }}_{i})+d_{\text{rSPR}}(T_{\rho },{T^{\prime }}_{\rho }).$$

## Conclusion

In this paper, we have shown that Hill et al's conjecture [1] and the underlying divide-and-conquer approach cannot be used to calculate the rSPR distance between two phylogenies exactly. To provide some intuition why this conjecture fails, consider the following. Let $\left(T_{\text{1}},{T^{\prime }}_{1}),...,(T_{t},{T^{\prime }}_{t}),(T_{\rho },{T^{\prime }}_{\rho }\right)$ be a cluster sequence with respect to Θ = *solvable *for two rooted binary phylogenetic trees T and $T^{\prime }$. Calculating a maximum-agreement forest for each tree pair ($T_{i},{T^{\prime }}_{i}$), taking their union, and, for each *i *∈; {1, 2, ..., *t*}, joining the element containing *a_i _*with the element containing *ρ_i _*can potentially result in a set, say G, which contains an element that is a subset of {*a*_1_, *a*_2_, ..., *a_t _*, *ρ*_1_, *ρ*_2_, ..., *ρ_t_*}. In the case of our counterexample,

$$G=\{\{1,2,3\},\{4,5,6\},\{7,8,9\},\{10,11,12\},\{13,14,15,\;\rho \},\{a_{1},\;a_{2},\;\rho_{1},\;\rho_{2}\}\}$$

contains one such element. Trivially, this element is not part of any agreement forest for T and $T^{\prime }$ while G - {{*a*_1_, *a*_2_, *ρ*_1_, *ρ*_2_}} is precisely a maximum-agreement forest for T and $T^{\prime }$. Consequently, a divide-and-conquer approach that exactly calculates $d_{\text{rSPR}}(T,T^{\prime })$ needs to take into account the number of elements in G that are subsets of {*a*_1_, *a*_2_, ..., *a_t _*, *ρ*_1_, *ρ*_2_, ..., *ρ_t_*}; otherwise, the result may be an overestimate of the exact solution. Alternatively, one can approach the problem by finding a strategy which guarantees that no element in G is a subset of {*a*_1_, *a*_2_, ..., *a_t _*, *ρ*_1_, *ρ*_2_, ..., *ρ_t _*}. This is the underlying idea of Theorem 4 which uses a slightly more restricted version of Hill et al's conjecture and finally gives the desired outcome. Hence, decomposing T and $T^{\prime }$ into a cluster sequence with respect to Θ = *subtree-like *can be used to speed up the exact calculation of $d_{\text{rSPR}}(T,T^{\prime })$.

However, for practical problem instances, it may be unlikely to find many subtree-like clusters. For example, the two phylogenies shown in Figure 1 do not have any common subtree-like cluster. This is due to the restricted definition of such a cluster which requires that a vertex whose set of descendants is a common cluster of two rooted binary phylogenetic *X*-trees T and $T^{\prime }$ has the same parent vertex than a common subtree of T and $T^{\prime }$. To lessen this problem, an alternative approach--that has recently been published by Linz and Semple [6]--can be applied. This paper describes a more general divide-and-conquer approach that exactly computes the rSPR distance between T and $T^{\prime }$ for when a cluster sequence $\left(T_{\text{1}},{T^{\prime }}_{1}),...,(T_{t},{T^{\prime }}_{t}),(T_{\rho },{T^{\prime }}_{\rho }\right)$ with respect to Θ = *minimal *for T and $T^{\prime }$ is given. Loosely speaking, the authors calculate a so-called minimum-weight partition G of *X *∪ {*ρ*} ∪ {*a*_1_, *a*_2_, ..., *a_t _*, *ρ*_1_, *ρ*_2_, ..., *ρ_t_*} such that G contains an agreement forest (not necessarily a maximum-agreement forest) for each tree pair ($T_{i},{T^{\prime }}_{i}$). To compute G, it has been shown that applying a 'bottom-up' approach which locally works on subtrees of each tree pair ($T_{i},{T^{\prime }}_{i}$) guarantees that the number of elements in G that are subsets of {*a*_1_, *a*_2_, ..., *a_t _*, *ρ*_1_, *ρ*_2_, ..., *ρ_t_*} is maximized while |G| is minimized.
